# A specific immune transcriptomic profile discriminates chronic kidney disease patients in predialysis from hemodialyzed patients

**DOI:** 10.1186/1755-8794-6-17

**Published:** 2013-05-10

**Authors:** Gianluigi Zaza, Simona Granata, Federica Rascio, Paola Pontrelli, Maria Pia Dell’Oglio, Sharon Natasha Cox, Giovanni Pertosa, Giuseppe Grandaliano, Antonio Lupo

**Affiliations:** 1 Renal Unit, Department of Medicine, University-Hospital of Verona, Piazzale A. Stefani 1, Verona 37126, Italy; 2 Renal, Dialysis and Transplant Unit-Department of Emergency and Transplantation, University of Bari, Piazza Giulio Cesare 1, Bari 70124, Italy; 3 Department of Medical and Surgical Sciences, University of Foggia, Via Pinto, 1, Foggia 71100, Italy

## Abstract

**Background:**

Chronic kidney disease (CKD) patients present a complex interaction between the innate and adaptive immune systems, in which immune activation (hypercytokinemia and acute-phase response) and immune suppression (impairment of response to infections and poor development of adaptive immunity) coexist. In this setting, circulating uremic toxins and microinflammation play a critical role. This condition, already present in the last stages of renal damage, seems to be enhanced by the contact of blood with bioincompatible extracorporeal hemodialysis (HD) devices. However, although largely described, the cellular machinery associated to the CKD- and HD-related immune-dysfunction is still poorly defined. Understanding the mechanisms behind this important complication may generate a perspective for improving patients outcome.

**Methods:**

To better recognize the biological bases of the CKD-related immune dysfunction and to identify differences between CKD patients in conservative (CKD) from those in HD treatment, we used an high-throughput strategy (microarray) combined with classical bio-molecular approaches.

**Results:**

Immune transcriptomic screening of peripheral blood mononuclear cells (1030 gene probe sets selected by Gene-Ontology) showed that 275 gene probe sets (corresponding to 213 genes) discriminated 9 CKD patients stage III-IV (mean ± SD of eGFR: 32.27±14.7 ml/min) from 17 HD patients (p < 0.0001, FDR = 5%). Seventy-one genes were up- and 142 down-regulated in HD patients. Functional analysis revealed, then, close biological links among the selected genes with a pivotal role of *PTX3*, *IL-15* (up-regulated in HD) and *HLA-G* (down-regulated in HD). ELISA, performed on an independent testing-group [11 CKD stage III-IV (mean ± SD of eGFR: 30.26±14.89 ml/min) and 13 HD] confirmed that HLA-G, a protein with inhibition effects on several immunological cell lines including natural killers (NK), was down-expressed in HD (p = 0.04). Additionally, in the testing-group, protein levels of CX3CR1, an highly selective chemokine receptor and surface marker for cytotoxic effector lymphocytes, resulted higher expressed in HD compared to CKD (p < 0.01).

**Conclusion:**

Taken together our results show, for the first time, that HD patients present a different immune-pattern compared to the un-dialyzed CKD patients. Among the selected genes, some of them encode for important biological elements involved in proliferation/activation of cytotoxic effector lymphocytes and in the immune-inflammatory cellular machinery. Additionally, this study reveals new potential diagnostic bio-markers and therapeutic targets.

## Background

Chronic kidney disease (CKD) is a major and growing challenge for health care systems. The prevalence rates of CKD appear to be increasing globally [1-3] primarily as a consequence of the augmented incidence of diabetes, hypertension, and aging population [4-6].

An international medical consensus has classified CKD into five stages according to the glomerular filtration rate (GFR) [7]. During the progression of renal damage, CKD patients undergo considerable metabolic changes, enhancement of inflammation/oxidative stress and significant development of several immunological alterations [8-11].

In particular, it has been well described that CKD patients develop a complex interaction between the innate and adaptive systems, in which immune activation (hypercytokinemia and acute-phase response) and immune suppression (impairment of response to infections and poor development of adaptive immunity) coexist [12]. In this setting, circulating uremic toxins and microinflammation play a critical role [13,14]. During inflammation, vasodilatation, vascular permeability, movement of inflammatory cells, and activation of cells of the immune system are increased [15,16].

In last stage of CKD (end stage renal disease), the enhancement of these clinical complications requires a rapid start of renal replacement therapies (RRTs) to ensure patients survival. Among RRTs, hemodialysis (HD) represents the most advanced form of artificial renal support. This method, through an extracorporeal circuit, removes waste products generated from normal metabolic processes, uremic toxins and normalizes body fluid and electrolytes [17].

Over the past five decades, the dialysis devices and membranes (dialysis filters) used for the treatment of CKD have continuously evolved and their basic structure has been modified to improve the biocompatibility profile of the material. This has led to the more effective removal of molecules involved in the development of complications associated with dialysis treatment [18-20]. Thus, the elaboration of biocompatible materials and the introduction of more effective pharmacological strategy to reduce microinflammation and oxidative stress are great challenges in nephrology.

However, although, during HD, we assist to an improvement of clinical status, patients undergoing this treatment still present important biological/biochemical alterations and they may develop serious clinical complications, such as amyloid arthropathy, bone loss diseases and accelerated atherosclerosis [21,22].

All these complications seem to be closely related to two key factors: a defective immune system function and continuous non-specific immune cell stimulation by dialysis devices [13].

In particular, several literature reports [10,23-25] demonstrated that HD patients present multifactorial biological and cellular dysfunctions including: activation of mononuclear cells, complement fixation, cytokine synthesis and release, reactive oxygen species (ROS), carbonyl stress and nitric oxide (NO) synthesis. However, HD may have positive effects on the immune-system. In particular, Kaul et al. have reported that initiation of HD led to a significant improvement of in vitro T-cell proliferation [26].

In the last years, several different therapeutic interventions have been proposed to prevent or at least limit HD-related cellular/biological alterations, including the use of biocompatible membranes that could limit the complement activation and cytokine release; the activation of high flux dialysis or hemofiltration that would reduce the circulating levels of complement-activating molecules and the accurate use of sterile water [18,27]. However, at the moment, we are still far from a total development of full biocompatible dialysis procedure.

The present study, utilizing an innovative transcriptomic approach combined to classical molecular methodologies, has been undertaken to better clarify biological mechanisms underlying immune dysfunction in uremic patients, the influence of HD on immune dysregulation and to identify new bio-molecular markers potentially useful as diagnostic tools and target for new therapeutic interventions.

## Methods

### Patients

A total of 50 subjects, after signing informed consent, were included in the study and divided in a training-group (n = 26) and a testing-group (n = 24).

A) Training-group

This population was used for the microarray analysis and to generate the initial hypothesis-generating genomic model. It included 9 CKD patients on stage III-IV (mean ± SD of estimated GFR by CKD-EPI formula: 32.27±14.7 ml/min) and 17 HD patients. This population was also used for RT-PCR experiments to validate microarray method. In addition, 8 healthy subjects (mean age±SD = 51.6±5.8 years and gender: 4 male and 4 female) were included as control (see Additional file 1: Figure S1A and B).

B) Testing-group

This group of patients was used to confirm and validate results obtained by microarray and it included 11 CKD on stage III-IV (mean ± SD of estimated GFR by CKD-EPI formula: 30.26±14.89 ml/min) and 13 HD patients.

All HD patients were stably treated, for at least 1 year, three times a week (4–5 hours per session), using synthetic membrane dialyzers. According to the modalities of the renal replacement therapy, 26 patients were treated with standard bicarbonate hemodialysis (n:15 in training-group and n:11 in testing-group) and 4 patients with on-line post-dilution hemodiafiltration (HDF) (n:2 in training-group and n:2 in the testing-group). For the HDF patients the substituted volume was approximately 100 ml/min. All patients presented a eKT/V value higher than 1.3. Moreover, based on several literature evidences [28,29] and to avoid additional confounding factors linked to the activation of the inflammatory machinery, all patients included in our study had native AV fistula.

During the study period, no CKD patients received dialysis treatment. In addition, all patients suffering from systemic autoimmune disorders, infectious diseases, diabetes, chronic lung diseases, neoplasm, or inflammatory diseases and patients receiving antibiotics, corticosteroids, or non-steroidal anti-inflammatory agents were excluded. The seven patients with diagnosis of glomerulonephritis as cause of CKD were affected by: Immunoglobulin A nephropathy (n:3), minimal change disease (n:2), focal segmental glomerulosclerosis (n:1) and membranous glomerulonephritis (n:1). No patients had symptomatic coronary artery diseases or a family history of premature cardiovascular diseases. Furthermore, there were some differences in the pharmacological treatment between HD and CKD patients. In particular, 86.6% of HD versus 60% of CKD patients were treated with recombinant Human Erythropoietin (rHuEPO); 63.3% of HD versus 5% of CKD patients were supplemented with intravenous (IV) iron; 40% of HD patients were treated with paracalcitol, while no CKD patients were treated with this drug. The main clinical and demographic characteristics of the subjects included in the training and testing group are summarized in Table 1. The study was carried out according to Declaration of Helsinki and approved by the institutional ethical board of the University Hospital “Policlinico di Bari”, Bari, Italy.

**Table 1:** Patient demographics and clinical characteristics

	**Training-group**			**Testing-group**		
	**CKD**	**HD**	**P.value**	**CKD**	**HD**	**P.value**
Number	9	17	/	11	13	/
Gender (M/F)	5/4	10/7	0.87	6/5	8/5	0.72
Age (years)	52.12 ± 5.39	52.86 ± 8.63	0.95	51.03 ± 7.59	50.32 ± 9.18	0.95
Cause of CKD: GN,ADPKD, renal vascular, desease, unknown	1,3,2,3	2,6,4,5	0.90	2,4,3,2	2,4,2,5	0.72
Time on dialysis(years)	/	4.98 ± 0.56	/	/	5.16 ± 0.75	/
BMI (kg/m^2^)	21.4 ± 1.16	21.8 ± 1.01	0.80	21.9 ± 0.82	21.5 ± 2.23	0.90
Systolic blood pressure (mmHg)	135 ± 13.22	136 ± 13.22	0.96	137 ± 9.62	137 ± 12.86	0.98
Diastolyic blood pressure (mmHg)	82 ± 9.43	84 ± 10.01	0.89	82.3 ± 9.85	86 ± 9.67	0.81
CRP (ng/ml)	2.65 ± 2.49	3.11 ± 3.45	0.92	2.32 ± 2.69	3.33 ± 3.62	0.85
Hemoglobin (g/dl)	11.13 ± 1.01	11.48 ± 1.98	0.90	11.86 ± 1.67	11.38 ± 1.85	0.87

GN: Glomerulonephritis; ADPKD: Autosomal dominant polycystic kidney disease; BMI: Body mass index; CRP: C reactive protein. Values are expressed as mean±SD. P-values calculated by *T*-Test.

### Lymphocyte subset count

For all subjects included in the study, circulating T lymphocyte subsets, Natural Killer (NK) cells and B lymphocytes were determined using CYTO-STAT tetraCHROME CD45-FITC/CD4-PE/CD8-ECD/CD3-PC5, CYTO-STAT tetraCHROME CD45-FITC/CD56-RD1/CD19-ECD/CD3-PC5 (Beckman Coulter) and the monoclonal antibodies CD16-PE (Beckman Coulter), CD3-FITC, TCR PAN gamma/delta. Flow-Count Fluorospheres were used to determine the absolute cell counts. Red blood cells were lysed with the COULTER ® ImmunoPrep™ Reagent System and the remaining white blood cells were analyzed by a Navios flow cytometry system with the Navios tetra Software package.

### Peripheral blood mononuclear cells (PBMCs) isolation

Twenty ml of whole blood were collected from all subjects included in both training- and testing-group. For HD patient the biological material was obtained at the beginning of the second HD session of the week. PBMC were isolated by density separation over a Ficoll-Paque™ (GE healthcare, Sweden) gradient (460 g for 30 min). PBMC were washed three times with PBS pH 7.4/1 mM EDTA (Sigma, Milan, Italy). Cells were then counted and their viability was assessed by trypan blue exclusion (>90% PBMC were viable).

### RNA extraction and gene expression profiling

For all subjects included in the training-group, total RNA was isolated by RNeasy mini kit Qiagen (QIAGEN AG, Basel, Switzerland) following manufacturer’s protocol. The RNA yield was evaluated with Nanodrop (ND-100 UV-1000 spectrophotometer) at 260 nm and total RNA integrity was assessed by electrophoresis using the Agilent 2100 Bioanalyzer (Agilent, Palo Alto, CA, USA). RNA was, then, processed and hybridized to the GeneChip Human Genome U133A (n = 5) and Plus (n = 29) oligonucleotide microarray (Affymetrix, Santa Clara, CA, USA). For our analysis, we used a dataset including 22,283 gene probe sets, representing 12,357 human genes and 3,800 ESTs (Affymetrix; see the manufacturer’s manual for detailed protocol). We used the default settings of Affymetrix Microarray Suite software version 5 to calculate scaled gene expression values. Results of the microarray experiments are available in Gene Expression Omnibus (Accession number GSE15072).

### Reverse transcription-polymerase chain reaction (RT-PCR)

Total RNA (1 μg) for the 26 patients included in the training-group was reverse transcribed with the Cloned AMV first-strand cDNA synthesis kit (Invitrogen) using Oligo(dT)_20_ (50 μM) and following the manufacturer’s instructions. PCR reactions were performed using primer designed by the aid of the Primer3 software (http://frodo.wi.mit.edu). For PTX3 mRNA expression, the primer sequences were: forward 5′-CATCCAGTGAGACCAATGAG-3′ and reverse 5′-GTAGCCGCCAGTTCACCATT-3′. The conditions of amplification were: 94°C for 20 sec, 58°C for 20 sec, 70°C for 20 sec for a total of 30 cycles of amplification. The primers used for IL-15 were forward 5′-GTATTGTAGGAGGCATCGTG-3′ and reverse 5′- CTCATTACTCAAAGCCACGG-3′. The conditions of amplification were: 94°C for 20 sec, 57°C for 20 sec, 70°C for 20 sec for a total of 30 cycles of amplification. β-actin PCR products were used as control gene. β-actin primer sequences were forward 5′-ggcatcgtgatggactccg-3′ and reverse 5′-gctggaaggtggacagcga-3′. The conditions of amplification were: 94°C for 20 sec, 65°C for 20 sec, 70°C for 20 sec for a total of 30 cycles of amplification. PCR products were electrophoretically separated on agarose gels and stained with ethidium bromide. The density of each band, corresponding to a specific PCR product, was densitometrically quantified by pixel density using NIH Image J image software http://rsb.info.nih.gov/ij/. The ratio between PTX3, IL-15 and β-actin PCR products were used as indexes of PTX3 and IL-15 gene expression.

### Elisa

Because of HLA-G gene was one of the identified genes by microarray, we decided to measure the soluble form of HLA-G levels in the plasma of all patients including in the testing-group. The assay was performed using commercially available enzyme-linked immunosorbent assay (ELISA) (BioVendor) according to the manufacturer’s instructions.

### Western blot analysis

Isolated PBMC from 6 CKD and 6 randomly selected HD patients were lysed in RIPA buffer (1 mM phenylmethylsulphonylfluoride, 5 mM EDTA, 1 mM sodium orthovanadate,150 mM sodium chloride, 8 μg/ml leupeptin,1.5% NonidetP-40, 20 mM Tris–HCl pH 7.4). The lysates were kept on ice for 30 min and centrifuged at 10000g at 4°C for 10 min. The supernatants were collected and stored at −80°C until use. Aliquots containing 50 μg of proteins from each lysate were subjected to SDS–PAGE and then electrotransferred onto PVDF membrane (Biorad). The blot was incubated with anti-CX3CR1 antibody (Abnova). The membrane was washed twice in TBS and incubated for one hour at RT with horseradish peroxidase-conjugated anti-rabbit IgG (Santa Cruz Biotechnology). The same membrane was stripped and immunoblotted again with anti-actin monoclonal antibody (Sigma) and with horseradish peroxidase-conjugated anti-mouse (Biorad). The ECL-enhanced chemiluminescence system (Amersham) was used for detection. Images were quantified by Image J1.34 Software (http://rsb.info.nih.gov/ij/). The intensity of band was normalized to the actin signal.

### Statistical analysis

Results were expressed as mean ± SD. *T*-test, ANOVA and Fisher’s exact test were used to assess differences in clinical and demographic features. A value of P < 0.05 was considered to be statistically significant.

We selected a total of 1030/22283 gene probe sets (corresponding to 645 genes) involved in immune response pathway according to Gene Ontology (GO, http://www.geneontology.org). Gene expression values for the 1030 probe sets, scaled to the target intensity of 2,500, were log transformed. *T*-TEST, Wilcoxon sum rank test and distinction calculation (DC) [30] were used to select probe sets discriminating the two groups of patients. False discovery rate (FDR) was estimated using an empirical Bayesian approach based on permutations (n = 500) and Storey’s q-value [31]. R 2.0.1 statistical software was used to perform the above analyses. Principal component analysis (PCA) and hierarchical clustering were performed using Spotfire DecisionSite 9.0 (http://www.spotfire.com). To assess the biological relationships among genes, we used the Ingenuity Pathway Analysis software (IPA, Ingenuity System, Redwood City, CA, USA; http://www.ingenuity.com). IPA is a knowledge database generated from the peer-reviewed scientific publications that enables discovery, visualization and exploration of functional biological networks in gene expression data and delineates the functions most significant to those networks. The 275 differentially expressed probe sets identified by microarray data, as discussed below, were used for network analyses. Affymetrix probe set IDs were uploaded into IPA and queried against all other genes stored in the IPA knowledge database to generate a set of networks. Each Affymetrix probe set ID was mapped to its corresponding gene identifier in the IPA knowledge database. Probe sets representing genes having direct interactions with genes in the IPA knowledge database are called ‘focus’ genes, which were then used as a starting point for generating functional networks. Each generated network is assigned a score according to the number of differentially regulated focus genes in our dataset. These scores are derived from negative logarithm of the P indicative of the likelihood of focus genes being found together in a network due to random chance. Scores of 4 or higher have 99.9% confidence level of significance. The resulting networks were represented in graphic format and adapted for publication.

## Results

### Analysis of the demographic and clinical features

For all patients included in the study, we did not find any difference in demographic and clinical features between CKD and HD patients (Table 1).

Analyzing results from lymphocyte subset count, we found that, although there were no differences in the percentage of each cell type between CKD versus HD patients, both groups showed significantly higher NK percentage compared to reference ranges (Table 2). This suggests that NK cells could have a key role in immune dysfunction in our CKD patients.

**Table 2:** Lymphocyte subset count distribution in both training- and testing-group

**Cellular type**	**Training-group**			**Testing-group**			
	**CKD(n = 9)**	**HD(n = 17)**	**p.value**	**CKD(n = 11)**	**HD(n = 13)**	**p.value**	**Ref**
Percent of Total CD3 + (T cells)	75.06 ± 10.52	75.65 ± 8.23	0.87	70.05 ± 8.38	74.80 ± 7.68	0.16	66-84
Percent of CD3+/CD4 + (Helper T cells)	51.34 ± 8.77	47.12 ± 12.18	0.36	41.46 ± 8.96	46.48 ± 8.69	0.17	43-55
Percent of CD3+/CD8+ (Supressor T cells)	22.47 ± 8.35	27.14 ± 9.62	0.23	25.94 ± 7.18	27.11 ± 8.44	0.72	20-32
Percent of CD3-/CD16 + CD56+ (NK cells)	17.49 ± 8.07	17.21 ± 8.53	0.93	21.59 ± 8.59	17.20 ± 7.33	0.19	4-10
Percent of CD3-/CD19+ (B cells)	7.33 ± 7.35	7.21 ± 3.45	0.28	8.45 ± 4.27	7.54 ± 2.64	0.52	6-12
CD4: CD8 Ratio	2.58 ± 1.02	2.08 ± 1.14	0.94	1.77 ± 0.70	2.00 ± 1/13	0.55	2-3

Values are expressed as mean±SD. P-values calculated by *T*-Test.

### Microarray analysis

**Figure 1:**
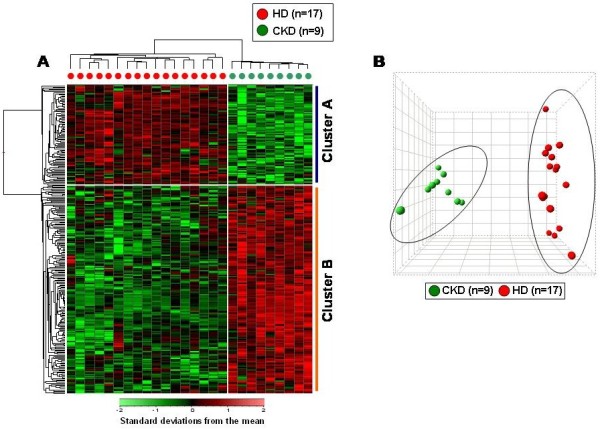
**“Supervised” hierarchical clustering and principal components analysis (PCA) discriminating chronic kidney disease (CKD) and hemodialysis (HD) treatment.** (**A**) Patients are depicted as vertical columns, with red symbols indicating HD (n = 17) and green indicating CKD (n = 9) patients. 275 gene probe sets were used for hierarchical clustering. The relative level of gene expression is depicted from lowest (green) to highest (red) according to the scale shown at the bottom. Cluster A and B include the top statistically significant up-regulated and down-regulated genes in HD compared to CKD, respectively. (**B**) PCA plot using the 275 selected gene probe sets discriminating the two groups of patients.

To identify an immunological transcriptomic profile able to discriminate CKD and HD patients, we analyzed the gene-expression profiling of 1030 gene probe sets (corresponding to 645 genes) involved in immune response in PBMC isolated from 17 HD and 9 CKD on stage III-IV K-DOQI. According to three independent statistical algorithms (*T*-test, Wilcoxon sum rank test and distinction calculation) and the estimate FDR, we identified 275 gene probe sets discriminating the two groups of patients (p < 0.0001, FDR = 5%). Among these gene probe sets, 89 (71 genes, Additional file 1: Table S1A) were up-regulated and 186 (142 genes, Additional file 1: Table S1B) were down-regulated in HD compared to CKD patients. The 2D hierarchical clustering using the 275 gene probe sets clearly separated patients into 2 distinct groups and PCA illustrates the degree of discrimination between the two groups of patients (Figure 1A and B).

Additionally, after including 8 healthy subjects (NORM) in the analysis, we found that our selected genes were also able to discriminate uremic patients in both conservative and hemodialysis treatment from healthy individuals (Additional file 1: Figure S1A and B). However, NORM group does not represent a valid control population because only matched with CKD and HD groups for few demographic features.

### Functional analysis of selected genes identified by microarray

**Figure 2:**
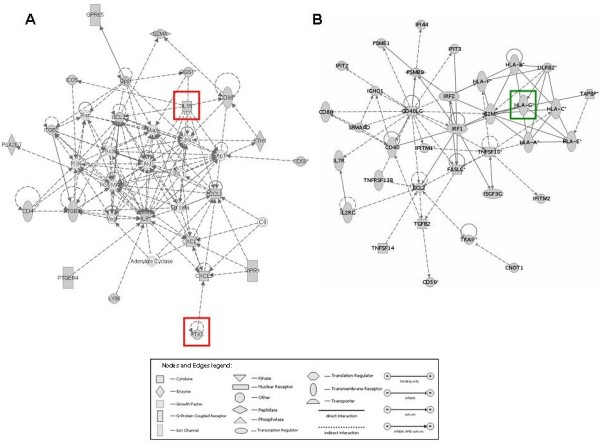
**Molecular networks generated by Ingenuity Pathway Analysis using the top selected genes discriminating chronic kidney disease (CKD) from hemodialysis (HD) patients.** Network, algorithmically generated based on the functional and biological connectivity among genes, was graphically represented as nodes (genes) and edges (the biological relationship between genes). Shaded nodes represented genes identified by our microarray analysis and others (empty nodes) were those that IPA automatically included because biologically linked to our genes based on evidence in the literature. Meaning of node shapes and edges are indicated. Red and green shaded genes were those used for validation by classical biomolecular techniques.

When the top selected genes up-regulated in HD patients were analyzed using the IPA software, we found that in the higher scored network (n = 44, p < 0.0001) were included genes encoding for biological elements primarily involved in chronic inflammation (*PTX3, IL-15, IL-8*), dendritic cells maturation (CD86), migration and adhesion of monocytes (CXCL3) (Figure 2A).

Additionally, in the higher scored network (n = 50, p < 0.0001) built by IPA using the top selected down-regulated genes in HD patients were included class I human leukocyte antigen genes (HLA-A, HLA-B, HLA-C, HLA-E, HLA-F, HLA-G) and those encoding activation cell surface molecules such as CD40L and FASL (Figure 2B). For the subsequent parts of the study, we focused on *PTX3*, *IL-15* and *HLA-G*.

### Validation of microarray using 2 representative genes involved in the immune response

**Figure 3:**
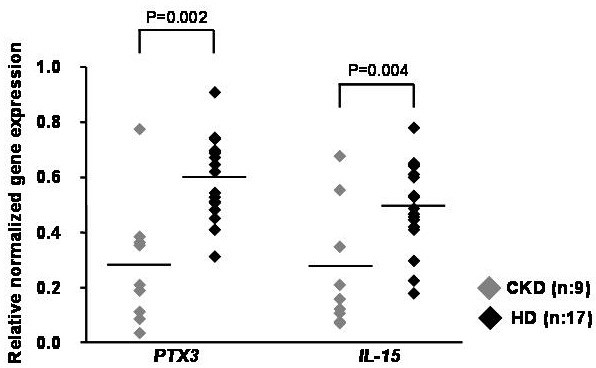
**PTX3 and IL15 gene expression in peripheral blood mononuclear cells by RT-PCR in chronic kidney disease (CKD) and hemodialysis (HD) patients.** Dot-plot represents PTX3 and IL15 gene expression assessed by RT-PCR in PBMC from 9 CKD and 17 HD patients. The bars indicate the median value. As depicted, HD patients showed significantly higher level of both genes compared to CKD patients. P values were calculated by *t*-test.

In order to validate microarray results, pentraxin 3 (PTX-3) and Interleukin-15 (IL-15) mRNA levels were measured in PBMC of all patients included in the training-group by PCR. These two genes encode for proteins primary involved in chronic inflammation. Both mRNA levels were significantly higher in HD compared to CKD patients (p < 0.01) (Figure 3). These results were in line with those obtained by the microarray analysis.

### Plasma HLA-G levels in testing-group

Among genes included in the most significant IPA network built using the significant down-regulated genes in HD, HLA-G has a pivotal role. This gene encodes for a protein with inhibitory properties towards major immune effector cells. In particular, it is able to inhibit the cytolytic activity and proliferation of NK cells.

**Figure 4:**
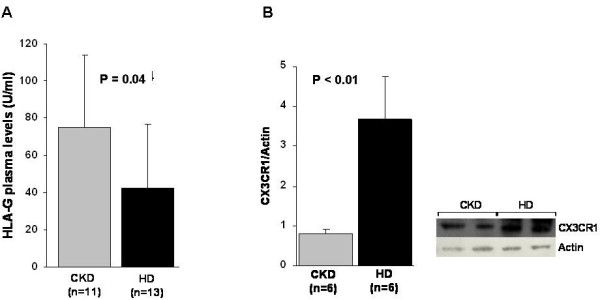
**HLA-G and CX3CR1 plasma protein expression by ELISA and Western blotting, respectively (A and B) in chronic kidney disease (CKD) and hemodialysis (HD) patients. A**) The histogram represents the mean ± SD of HLA-G levels assessed by ELISA in 11 CKD and 13 HD patients. HD patients presented significantly lower HLA-G level compared to CKD (p = 0.04); **B**) Histogram represents the mean ± SD of CX3CR1 protein level in total cell lysates of 6 CKD and 6 HD patients. CX3CR1 levels were significantly higher in HD compared to CKD patients (p < 0.01). On the right it is reported a representative western blotting experiment. P values were calculated by *t*-test.

Data analysis showed that HD patients had significantly lower HLA-G plasma level compared to CKD (p = 0.04) (Figure 4A).

### CX3CR1 protein expression

Since HD patients showed down-regulation of HLA-G, high level of IL-15 (a cytokine that stimulates NK proliferation and cytotoxicity), and inflammatory status as suggested by elevated level of PTX3, we decided to evaluate protein expression of chemokine receptor CX3CR1. CX3CR1 is a highly selective chemokine receptor and surface marker for cytotoxic effector lymphocytes (such as NK cells) and it seems to have a central role in several inflammatory diseases.

As shown in Figure 4B, CX3CR1 levels were significantly higher in HD compared to CKD patients (p < 0.01).

## Discussion

Over the years several studies have tried to clarify the biological machinery involved in both innate and adaptive immune dysfunction in CKD patients in conservative and HD treatment.

The causes of this condition seem to be primarily related to: a) an abnormal accumulation of pro-inflammatory cytokines as a consequence of decreased renal elimination and/or increased generation of uremic toxins; b) elevated oxidative stress, c) volume overload and d) augmented rate of sepsis [10,11].

Additionally, CKD-related immune dysfunction seems to be characterized by the coexistence of immune activation and immune suppression [12]. In this setting, circulating uremic toxins and microinflammation play a critical role. However, the exact intricate mechanism associated to the immune alterations in CKD need to be more elucidated.

To better understand the biological bases underlying the immune alterations in patients with severe renal damages and to assess differences between CKD patients in pre-dialysis and those undergoing HD treatment, we used, for the first time, a transcriptomic strategy combined with classical bio-molecular approaches. This methodology allowed us to reduce biases due to the relatively small number of patients enrolled because of highly selective inclusion/exclusion study criteria (e.g., good demographic/clinical matching, exclusion of under-dialyzed patients) and to minimize confounding factors.

Furthermore, our study was performed using an oligonucleotide microarray technology able to evaluate simultaneously the expression of more than 15,000 genes. However, to take full advantage of the opportunities offered by this high throughput method, it is necessary to manage, integrate and interpret a huge amount of data correctly. Thus, we decided to use a pathway analysis to focus our research on candidate genes known to be associated with immune response in order to reduce the false positive rate and the puzzling factors not directly associated with the aims of our research.

Microarray analysis revealed that 213 immunological genes were able to discriminate CKD from HD patients. In particular, 71 genes resulted up-regulated and 142 down-regulated in HD compared to CKD. Although these differences were primarily related to the transcriptomic changes induced by the dialysis treatment, we cannot exclude that the different pharmacological treatments may have contributed to increase the immune-transcriptomic differences between CKD and HD patients. In fact, as extensively described [32-35], some medications (e.g., rHuEPO, VDR activators and IV iron), may influence both inflammatory and immune response. It is plausible that the deregulation of some immunological genes in our HD patients (for example the up-regulation of CD86 and the down-regulation of CD40/CD40LG) may have been, at least partially determined by the chronic use of rHuEPO and/or paracalcitol. However, at the state of art, there is a lack of pharmacogenomics study in this field. Further studies are warranted to address this important point.

Interestingly, the biological network generated by Ingenuity Pathway Analysis software using the top HD up-regulated genes, showed that pentraxin 3 (*PTX3*) and Interleukin 15 (*IL-15*) had an important functional role. *PTX3* encodes for an acute phase protein produced by monocytes, macrophages and endothelial cells [36] and, as reported by several reports, its expression is high in plasma of patients with sepsis [37], acute myocardial infarction [38] and severe atherosclerosis [39].

Our results, confirming previous reports [40,41], indicate that HD enhances PTX3 levels and point out that this inflammatory biomarker may be a valuable candidate to measure chronic microinflammatory state in our nephrology/dialyzed patients. In fact, in our subset of patients, C reactive protein (CRP) was not different between CKD and HD probably because less sensitive and specific biomarker for this patient population.

Then, we observed that *IL-15* was highly expressed in our HD patients compared to CKD. This gene encodes for a 13 kDa cytokine produced by macrophages and other cell types in response to inflammatory and infective stimuli that shares many biological activities with IL-2 inducing the activation of JAK/STAT pathway and stimulating T and NK cells survival, proliferation and activation [42-45].

On the other hand, among the genes down-regulated in HD patients, we identified those encoding for the Human Leukocyte Antigen (HLA)-G. The HLA-G primary transcript encodes a transmembrane protein with a molecular weight of 39 kDa that may be classified as a nonclassical major histocompatibility complex class I molecule that differs from other HLA class I molecules with regard to its low polymorphism, restricted tissue distribution, slow turnover, immunosuppressive properties and limited peptide diversity [46-48].

Under physiological conditions, the production of HLA-G protein is restricted to trophoblast [49], thymic epithelial cells [50], first-trimester placental chorionic blood vessel endothelial cells [51], and IFN-γ-treated mononuclear phagocytes [52].

However, the up-regulation of this protein can be detected in several pathological conditions such as transplantation, tumors, viral infections and autoimmune diseases [53-57].

HLA-G gene expression is tightly regulated at both the transcriptional and post-transcriptional levels and through epigenetic mechanisms, which include DNA methylation and histone deacetylation [58]. In addition, different researchers have shown that HLA-G mRNA expression does not systematically result in the presence of the protein [59,60]. This implies a tight post-transcriptional regulation of HLA-G gene expression.

HLA-G possesses the capability to bind inhibitory receptors such as the Immunoglobulin-like transcript-2 and −4 (ILT2, ILT4) and the Killer Immunoglobulin-like Receptor (KIR)2DL4/CD158d with inhibitory effects [61,62].

In particular, HLA-G may have a direct immune-inhibitory function through blocking effector cells and indirect immune-inhibitory activity by regulatory cell generation. Via the direct inhibitory functions, HLA-G is able to inhibit the cytolytic activity and proliferation of NK [63], the antigen-specific cytolytic functions of α/β and γ/δ T lymphocytes [63,64], the alloproliferative response of T cells [65,66], the proliferation of NK and T cells [66] and the DCs maturation [67]. Therefore, the lower HLA-G expression in HD patients suggests that innate immune effector cells could be activated in this subset of patients confirming some reports showing that long-time dialysis enhances NK cytotoxic activity [68,69].

Furthermore, since HD patients showed down-regulation of HLA-G, high level of IL-15, and inflammatory status as suggested by increased level of PTX3, we decided to evaluate protein expression of chemokine receptor CX3CR1.

CX3CR1 is the fractalkine (CX3CL1) receptor and is expressed in cytotoxic effector lymphocytes such as NK, cytotoxic T lymphocytes (CTL) and γ/δ T cells which possess high levels of intracellular perforin and granzyme B [70] suggesting that its activation could be involved in migration of the cells to sites of inflammation [71]. Moreover, this receptor is up-regulated during chronic inflammation [72] and it is increased in monocytes, T lymphocytes and NK cells present in severe atherosclerosis and ruptured coronary plaques in patients with unstable angina pectoris [73].

As shown in Figure 4B, CX3CR1 levels were significantly higher in HD compared to CKD patients (p < 0.01).

## Conclusions

Although with the presence of some limitations, such as small number of patients included in the microarray analysis, impossibility of controlling all variables potentially able to influence the transcriptomic profile (e.g., etiological cause of CKD, drugs, diet) and absence of clinically matched healthy subjects, our study shows, for the first time, that HD patients present a different immune-pattern compared to CKD patients in conservative treatment. Among the selected genes, some of them encode for important biological elements involved in proliferation/activation of cytotoxic effector lymphocytes and in the immune-inflammatory cellular machinery. Additionally, this study reveals new potential diagnostic bio-markers and useful targets for innovative therapeutic interventions.

## Competing interests

The authors declare that they have no competing interests.

## Authors’ contribution

GZ, SG and GG designed research; GZ, SG, FR, MPD, SC and PP conducted research; GZ, SG, GG, GP and AL wrote the paper; GZ had primary responsibility for final content. All authors read and approved the final manuscript.

## Pre-publication history

The pre-publication history for this paper can be accessed here:

http://www.biomedcentral.com/1755-8794/6/17/prepub

## Supplementary Material

Additional file 1 Supplemental information. Click here for file
