# Supplementary material for: A specific immune transcriptomic profile discriminates chronic kidney disease patients in predialysis from hemodialyzed patients
Source: BMC Med Genomics. 2013 May 10;6:17. doi: 10.1186/1755-8794-6-17 (PMC3655909; doi:10.1186/1755-8794-6-17)
Supplement: Additional file 1 — Supplemental information. [file 1755-8794-6-17-S1.doc]

**Supplemental information for:**

**A SPECIFIC IMMUNE TRANSCRIPTOMIC PROFILE DISCRIMINATES CHRONIC KIDNEY DISEASE PATIENTS IN PREDIALYSIS FROM HEMODIALYZED PATIENTS.**

Gianluigi Zaza^1^, Simona Granata^1^, Federica Rascio^2^, Paola Pontrelli^2^, Maria Pia Dell’Oglio^2^, Sharon Cox^2^ , Giovanni Pertosa^2^ , Giuseppe Grandaliano^3^ , Antonio Lupo^1^

^1^ Renal Unit, Department of Medicine, University-Hospital of Verona, Verona, Italy

^2^ Renal, Dialysis and Transplant Unit­-Department of Emergency and Transplantation, University of Bari, Italy

^3^ Department of Biomedical Sciences, University of Foggia, Foggia, Italy

**Address correspondence to:**

Gianluigi Zaza, MD, PhD

Renal Unit, Department of Medicine,

University-Hospital of Verona, Verona, Italy.

Piazzale A. Stefani 1, 37126, Verona (VR), Italy

Tel. 045.8122528;

fax 045.8027311

E mail: [gianluigi.zaza@univr.it](mailto:gianluigi.zaza@univr.it)

**Figure 1S: (A) 2D Hierarchical clustering and (B) principal component analysis (PCA) discriminating healthy subjects (8 NORM), chronic kidney disease (9 CKD) and hemodialysis (17 HD) treatment.**

**Table A: Top discriminating genes up-regulated in HD compared to CKD patients.**

Listed are the 89 gene probe sets (71 genes) up-regulated in HD compared to CKD patients, selected according to three independent statistical algorithms (T-test, Wilcoxon sum rank test and distinction calculation) and the estimate FDR.

**Table B: Top discriminating genes down-regulated in HD compared to CKD patients.**

Listed are the 186 gene probe sets (142 genes) down-regulated in HD compared to CKD patients, selected according to three independent statistical algorithms (T-test, Wilcoxon sum rank test and distinction calculation) and the estimate FDR.
